# Cost-effectiveness thresholds used in the United States vs most favored nations

**DOI:** 10.1093/haschl/qxag081

**Published:** 2026-04-03

**Authors:** Hanxuan Yu, Peter J Neumann, David D Kim, Joshua T Cohen, Ashley A Leech

**Affiliations:** Center for Health Decision Science, Harvard University T.H. Chan School of Public Health, Boston, MA 02115, USA; Center for the Evaluation of Value and Risk in Health, Tufts University School of Medicine, Boston, MA 02111, USA; Department of Medicine and Public Health Sciences, Biological Sciences Division and the College, The University of Chicago, Chicago, IL 60637, USA; Center for the Evaluation of Value and Risk in Health, Tufts University School of Medicine, Boston, MA 02111, USA; Department of Health Policy, Vanderbilt University School of Medicine, Nashville, TN 37203, USA

**Keywords:** Cost-effectiveness analysis, Cost-effectiveness thresholds, Most favored nations

## Abstract

**Objectives:**

Cost-effectiveness thresholds inform whether health interventions represent good value for money, yet their use varies across countries. This study compares thresholds cited in published cost-effectiveness analyses (CEAs) in the United States with those in countries designated as the Most Favored Nations (MFNs) under the 2025 President's Executive Order on prescription drug pricing.

**Methods:**

We analyzed 6876 cost-per-QALY studies published between 1979 and 2023 from the Tufts CEA Registry. We standardized thresholds as multiples of each country's GDP per capita, and used logistic regression to estimate the probability of citing a threshold >1 × GDP per capita, adjusting for region, intervention type, disease area, and study period.

**Results:**

Over time, MFN studies shifted toward citing lower thresholds, whereas US thresholds consistently cited thresholds >1 × GDP per capita. After adjusting for other factors, MFN studies were less likely to cite higher thresholds than US studies. Cancer-related CEAs and CEAs of pharmaceutical interventions were more likely to cite higher thresholds.

**Conclusions:**

CEAs in the United States and peer high-income nations cite remarkably different thresholds, with MFNs citing lower value benchmarks over time. Policymakers should be cautious about adopting pricing policies that would implicitly subject US pharmaceutical spending to benchmarks developed in different institutional and fiscal contexts.

## Introduction

In November 2025, the Center for Medicare & Medicaid Services (CMS) announced a new drug payment scheme, the “GENErating cost Reductions fOr U.S. Medicaid” (GENEROUS) Model,^1^ which operationalized the Trump Administration's executive order entitled “Delivering Most-Favored-Nation Prescription Drug Pricing to American Patients.^2^” The order calls on the Department of Health and Human Services (HHS) to “communicate most-favored-nation (MFN) price targets to pharmaceutical manufacturers to bring prices for American patients in line with comparably developed nations.^2^ “The MFN price would reflect pricing in the other six G-7 countries (United Kingdom, France, Germany, Italy, Canada, and Japan), Denmark, and Switzerland.^1,3^

The Executive Order and the GENEROUS Model aim to ensure that Americans pay no more for prescription drugs than consumers in other countries by aligning prices across markets, most directly through reductions in US prices. In principle, this alignment could also be achieved by increasing prices paid in MFNs or through other mechanisms, such as by restricting the availability of drugs in MFNs. In cases where manufacturers align prices by reducing what they charge in the United States, the result may be the “importation” of pharmaceutical pricing criteria used in the MFN nations.

This comparison raises questions about how the criteria for the United States and MFNs may differ. The question is important because even if the Executive Order reduces prices in the United States, an ostensible benefit, it is unclear what this could mean for drug manufacturers and investment in future innovation^4-7^ In short, aligning US pricing criteria with MFN benchmarks could influence population health by reducing drug prices and improving affordability/access, while also altering resource allocation in ways that may affect incentives for future drug development.^8,9^

To assess the potential impact of aligning US pharmaceutical pricing criteria with those used in MFNs, we examined how one criterion, used in cost-effectiveness analyses, differs across settings. We do so by comparing value benchmarks cited in cost-per-quality-adjusted life year (QALY) analyses set in both the United States and in MFNs. Value benchmarks, also known as “thresholds,” reflect judgments made by study authors or Health Technology Assessment (HTA) bodies about where to draw the dividing line between cost-effectiveness ratios considered favorable (interventions that deliver QALYs at a cost below the threshold) and those considered unfavorable (ratios exceeding the threshold).

Examining how these benchmarks differ across countries helps contextualize how health systems evaluate whether healthcare spending provides sufficient health gains relative to cost. A finding that MFN cost-effectiveness analyses (CEAs) report lower (ie, more stringent) thresholds than US studies may imply that drug spending in those countries could impose a higher opportunity cost than in the United States. As a result, additional drug spending is evaluated in those countries under tighter budget constraints. We further explored how these thresholds may be changing over time. Second, to further investigate how pricing criteria may differ both within and across countries, we examined whether benchmarks depend on the type of intervention (pharmaceuticals vs other interventions) and the disease area (cancer vs non-cancer).

## Methods

We conducted two analyses.

**Analysis 1—Threshold distribution overall and over time**: We described the distribution of cost-effectiveness thresholds reported by cost-per-QALY studies for the United States and for MFNs. This analysis reported both the distribution across all CEAs and how these distributions changed over time.

**Analysis 2—Factors influencing the threshold distribution**: We used logistic regression to analyze the relationship between the threshold values cited and the following putative predictive factors: region (MFN vs United States), intervention type (pharmaceutical vs other), disease area (cancer vs non-cancer, identified by ICD 10 code C00-D48), and cost year. While the “non-cancer” category encompasses a heterogeneous set of interventions, our objective was to compare cancer CEAs with all other CEAs because prior work suggests that cancer interventions are often evaluated using higher cost-effectiveness thresholds.^10^ We therefore examined whether this pattern persists across MFN countries and the United States. We assessed statistical significance at the 0.05 level and used R version 4.4.1.

### Data source

We used the Tufts Medical Center CEA Registry, a comprehensive database of English-language published CEAs that report incremental cost-per-QALY ratios across diseases and treatments. For each published study, the Registry records the cost-effectiveness threshold(s) the authors used to categorize an intervention's cost-effectiveness as either favorable, suggesting that the intervention satisfies a country's “value-for-money” criterion (CE ratio falls below the threshold), or as unfavorable, suggesting that the intervention does not satisfy this criterion (ratio exceeds the threshold). Predictor variables for the regression (region, cost year, intervention type, and disease area) also came from the Tufts Medical Center CEA Registry.

### Inclusion and exclusion criteria

We limited attention to studies from the United States and from eight MFNs referenced by the GENEROUS Model, including the United Kingdom, France, Germany, Italy, Canada, Japan, Denmark, and Switzerland. We included articles published from 1979 to 2023, with reported cost years no later than 2022. We excluded studies reporting thresholds in currencies not tracked by the World Bank or in uncommon formats (eg, producer price index-based thresholds).

### Outcomes

For the first analysis, the outcome was the threshold value (the monetary value of a QALY) as a multiple of GDP per capita, categorized as ≤1 × GDP per capita, 1-3 × GDP per capita, and >3 × GDP per capita. We standardized reported threshold values as follows: (1) For thresholds reported in non-USD currencies, we converted the value to USD using World Bank exchange rates for the study's cost year; and (2) inflated or deflated this value to the World Bank reference year (2015 USD) using the personal consumption expenditures (PCE) price index; here, we used the PCE price index to align with the World Bank's approach for internationally comparable adjustments. Previous studies have shown that the PCE and personal health care [PHC] produce close results.^11^ If the original source already reported a cited threshold value as a multiple of GDP per capita, no currency standardization was required. For all other thresholds, we expressed reported cost-effectiveness thresholds as GDP-per-capita multiples by dividing each threshold by the country's GDP per capita in the study's cost year, with both values converted to a common reference-year USD. For thresholds (in all formats) reported as ranges, we used the range's upper bound. For studies referring only “the National Institute for Health and Care Excellence (NICE) thresholds”, which have long been set at £20 000–£30,000, we assumed a value of £30 000 per QALY. We coded articles that did not report a threshold as a separate category designated “No threshold cited” Unless noted, our analyses limit attention to studies reporting a threshold.

For the second analysis, the binary outcome depended on whether the GDP-normalized threshold exceeded 1 × GDP per capita. We tested the association between this outcome and CEA region, intervention type, disease area, and cost year. We categorized the cost year into four periods (before 2000, 2000-2009, 2010-2019, and 2020 or later) to differentiate trends across time periods, with the period before 2000 as the reference category. We estimated both the logistic regression coefficient for the predictor and estimated marginal means, which represent the average predicted outcome values (ie, probabilities in a logistic regression) for a given factor level or group, adjusted for the other variables in the model.

As scenario analyses, we conducted country-level summary statistics, a logistic regression model with country fixed effects, and a weighted decomposition analysis (multiplying each country's estimated effects by its share of MFN studies), to evaluate whether the MFN– United States difference reflects broad cross-country heterogeneity (Appendix 1). We also performed subgroup-specific analyses restricted to CEAs involving pharmaceutical interventions to better align with the scope of the GENEROUS model (Appendix 2) and estimated a multinomial logistic regression model that treated “No threshold cited” as a separate category to assess the impact of missing data (Appendix 3).

## Results

We identified 3388 MFN CEAs and 3488 U.S. CEAs; 633 MFN studies (18.7%) and 627 U.S. CEAs (18.0%) did not report a threshold (Table 1). The median threshold value was 1.28 × GDP per capita ($110 000 per QALY in 2024 USD, or £51 000 per QALY in 2024 GBP), and the mean value was 1.70 × GDP per capita ($146 000 per QALY in 2024 USD, or £68 000 per QALY in 2024 GBP).

**Table 1.** Thresholds cited in cost-effectiveness analyses.

	Cost Year Number of studies (column percentage)				
	Before 2000	2000—2009	2010—2019	Since 2020	All periods
**Most Favored Nations**	154	1163	1951	120	3388
≤1 × GDP per capita	14 (9.1)	235 (20.2)	849 (43.5)	57 (47.5)	1155 (34.1)
1-3 × GDP per capita	30 (19.5)	623 (53.6)	796 (40.8)	46 (38.3)	1495 (44.1)
>3 × GDP per capita	13 (8.4)	36 (3.1)	54 (2.8)	2 (1.7)	105 (3.1)
Any thresholds	57 (37.0)	894 (76.9)	1699 (87.1)	105 (87.5)	2755 (81.3)
No threshold cited	97 (63.0)	269 (23.1)	252 (12.9)	15 (12.5)	633 (18.7)
**United States**	380	1016	1875	217	3488
≤1 × GDP per capita	11 (2.9)	130 (12.8)	496 (26.5)	28 (12.9)	665 (19.1)
1-3 × GDP per capita	116 (30.5)	622 (61.2)	1096 (58.4)	164 (75.6)	1998 (57.3)
>3 × GDP per capita	61 (16.1)	47 (4.6)	77 (4.1)	13 (6.0)	198 (5.7)
Any thresholds	188 (49.5)	799 (78.6)	1669 (89.0)	205 (94.5)	2861 (82.0)
No threshold cited	192 (50.5)	217 (21.4)	206 (11.0)	12 (5.5)	627 (18.0)

Source: Author's analysis of published cost-effectiveness analyses (CEAs) from the Tufts Medical Center's CEA Registry database.

Table 1 summarizes cost-effectiveness thresholds cited in cost-per-QALY studies from the CEA registry, categorized as ≤1 × GDP per capita, 1-3 × GDP per capita, and >3 × GDP per capita, by cost year group and region. The table also includes studies with no threshold cited, which are comparatively more common before 2000.

### Analysis 1 results

**Figure 1.**
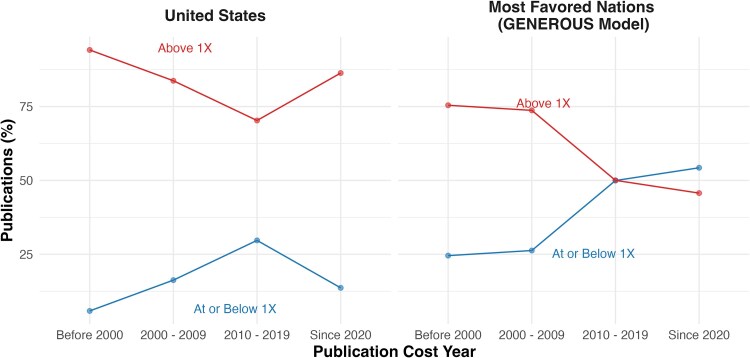
Percent of cost-effectiveness analyses citing a threshold above benchmark (1 × GDP per capita), by region. Source: Author's analysis of published cost-effectiveness analyses (CEAs) from the Tufts Medical Center's CEA Registry database. *Note.* Figure 1 shows how the proportion of CEAs citing thresholds above vs at or below the benchmark (1 × GDP per capita) has changed over time (excluding studies with no reported threshold), with U.S. studies increasingly using higher thresholds while MFN studies have shifted toward lower thresholds.

Figure 1 illustrates how the distribution of cited CEA thresholds has changed over time in both MFNs and in the United States. Among MFN studies, the proportion citing thresholds at or below 1 × GDP per capita benchmark increased over time and became the majority in the recent post-2020 period. In contrast, US studies consistently cited thresholds above the 1 × benchmark across all periods, with only a temporary rise in the share citing lower thresholds. Results restricted to pharmaceutical CEAs are consistent with the main findings (Appendix Table S2). Because both regions exhibit substantial missingness before 2000 (United States: 50.5%; MFNs: 63.0%, as detailed in Table 1), the time trend is more reliably interpreted from 2000 onward.

### Analysis 2 results

Logistic regression (Table 2) showed: (1) MFN CEAs were less likely than US CEAs to cite thresholds exceeding 1 × GDP per capita. Country-level regression results (Appendix 1) show a similar pattern of lower threshold use across MFNs. Although the UK and Canada account for most MFN studies in the sample, all MFNs exhibited negative coefficients relative to the United States, indicating a consistent pattern of citing lower thresholds. (2) Pharmaceutical CEAs are more likely to cite thresholds exceeding 1 × GDP per capita. (3) CEAs for cancer interventions are more likely to cite thresholds exceeding 1 × GDP per capita, and (4) CEAs published during 2000-2009, 2010-2019, or since 2020 are all less likely to cite ratios exceeding 1 × GDP per capita than CEAs published before 2000. The multinomial regression that includes “No threshold cited” yields a similar pattern (Appendix 3).

**Table 2.** Logistic regression of citing cost-effectiveness threshold above 1 × GDP per capita.

Characteristics	Coefficients	SE	EMMs^a^	EMM difference (SE)
MFNs vs U.S.	−1.7692***	0.4511	MFNs: 0.683 U.S.: 0.884^b^	−0.201 (0.0239)
Cancer-related CEAs vs Non-cancer	0.8378***	0.0886	Cancer: 0.860 Non-cancer: 0.727	0.133 (0.0129)
Pharmaceutical Intervention vs Other	0.3178***	0.0635	Pharm: 0.826 Other: 0.775	0.0505 (0.0103)
Cost Year: Before 2000 (Reference)			MFNs: 0.793 U.S.: 0.957	−0.1654 (0.0551)
2000-2009	−1.1180***	0.3274	MFNs: 0.795 U.S.: 0.880	−0.0851 (0.0168)
×Region^c^	1.1313*	0.4685		
2010-2019	−2.0109***	0.3174	MFNs: 0.568 U.S.: 0.750	−0.1827 (0.0166)
×Region	0.9411*	0.4574		
Since 2020	−1.2023**	0.3749	MFNs: 0.524 U.S.: 0.871	−0.3469 (0.0553)
×Region	−0.0436	0.5322		

Source: Author's analysis of published cost-effectiveness analyses (CEAs) from the Tufts Medical Center's CEA Registry database.

Interaction terms between disease area and region and between intervention type and region were not significant.

Abbreviations: EMM, Estimated Marginal Mean; MFNs, Most Favored Nations; SE, Standard Errors.

^a^An Estimated Marginal Mean (EMM) is the model-based average predicted outcome (ie, probability in logistic regression) for a given factor level or group, adjusted for the other variables in the model.

^b^Interpretation: These are the predicted probabilities that a threshold exceeds 1 × GDP per capita after adjustment for other variables. 88.4% of US CEAs are predicted to cite thresholds exceeding 1 × GDP per capita, whereas the estimated probability that MFN thresholds exceed this value is 0.683. In short, US CEAs are more likely (by 20.1% points) to use thresholds above 1 × GDP per capita than MFNs.

^c^Interaction terms between cost year level (eg, for here 2000—2009) and region.

^*^: *P* < 0.05; **: *P* < 0.01; ***: *P* < 0.001.

## Discussion

Holding region (MFN vs United States), intervention type (pharmaceutical vs other), and disease area (cancer vs non-cancer) constant, cited GDP-normalized CEA thresholds are declining over time. That aggregate trend conceals an important difference between the United States and MFNs, however. Among MFN studies, controlling for intervention type and disease area, the probability of citing a threshold exceeding 1 × GDP per capita declined steadily since 2000 and fell below 50% in the post-2020 period. In contrast, US studies consistently cited thresholds above 1 × GDP per capita across all periods, with little evidence of a downward shift over time. In short, even after holding intervention type and disease area constant, US studies continue to reference higher threshold values over time, while MFNs have shifted toward citing lower threshold values.

The United States spends approximately twice as much on healthcare as a share of GDP as other high-income countries, despite similar levels of health care utilization.^12^ This difference is driven largely by differences in prices.^12^ In the United States, pharmaceutical manufacturers generally have greater flexibility in setting prices, subject to certain restrictions (eg, Medicaid “best price” legislation and Medicare negotiated prices mandated by the Inflation Reduction Act). Unlike many other high-income countries that rely on formal negotiation mechanisms and centralized HTA bodies, the United States lacks a single national authority that directly links prices to value assessment. However, non-governmental organizations, such as the Institute for Clinical and Economic Review (ICER), conduct value assessments that use cost-effectiveness thresholds, which have been considered by US payers when making coverage and formulary decisions. Survey evidence suggests that nearly 60% of US payers have consulted CEAs in price negotiations or reimbursement decisions.^13^ In 2018, CVS Health (one of the US largest Pharmacy Benefit Managers) experimented with referencing ICER's $100 000 per QALY benchmark in its formulary evaluations.^14^ In contrast, many peer countries tend to use value assessment (eg, CEAs) and other means to limit the prices manufacturers can charge. A consequence of the Trump administration's MFN policy would be closer alignment of US and MFN prices, implicitly aligning US spending criteria with value benchmarks reflected in MFNs.

Our analysis further shows that thresholds vary by intervention type and disease area. Authors were more inclined to cite higher thresholds for pharmaceuticals than for other types of interventions, possibly reflecting the pricing context in which many CEAs are conducted. In the United States, branded drugs, protected by patents and temporary market exclusivity, allow manufacturers to charge higher prices, which may result in higher incremental cost-effectiveness ratios.^15^ As a result, authors evaluating pharmaceutical interventions may reference higher thresholds commonly cited in the literature when interpreting cost-effectiveness results. Similarly, cancer-related CEAs also cited higher thresholds, consistent with previous findings suggesting that cancer care often receives greater priority in resource allocation^16-18^

Cost-effectiveness thresholds serve a dual purpose: they indicate society's willingness to pay for health improvements and provide a benchmark for identifying investments that yield lower returns than alternative spending opportunities. The debate over what the value of this benchmark should be is ongoing. Rising incomes should reduce the marginal value of additional consumption, and hence the monetary value of health should increase. On the other hand, healthcare opportunity costs suggest lower benchmark values. Empirical estimates of healthcare opportunity costs that reflect how much health the UK's National Health Service (NHS) typically produces suggest threshold values at or below £15 000/QALY^19-22^ These values fall well below NICE's longstanding £20 000-£30 000 threshold range, not to mention the recently proposed range of £25 000-£35 000 per QALY.^23^ In the United States, by comparison, ICER has cited benchmarks of $100 000-$150 000/QALY,^19^ values exceeding those cited by most peer nations even after adjustment for per-capita GDP.

This study has limitations. First, unlike formal thresholds codified in national HTA systems, the thresholds in our dataset reflect choices made by individual study authors. The degree of alignment between academic reporting and policy application likely varies by country. In contrast, agencies such as NICE or Canada's Drug Agency anchor reimbursement to threshold ranges; most US payers do not. Nonetheless, this perspective reflects the collective behavior and evolving norms of the research community and hence indicates the value placed on health in various countries.^20^

Second, while normalizing thresholds using GDP per capita facilitates comparability, it necessarily excludes thresholds based on other indices (eg, producer price index) or currencies not tracked by the World Bank, slightly reducing the representativeness of our sample.

Third, we observed a high level of missingness (“No threshold cited”) before 2000 and a smaller sample size after 2020. However, treating “No threshold cited” as a separate category in a sensitivity analysis (Appendix 3) did not alter our overall findings. Analysis 2 showed that the trend toward citing higher thresholds in the United States and lower thresholds in MFNs is evident even in the earliest (Before 2000) and most recent (Since 2020) periods. In addition, many studies published since 2020 rely on earlier cost years, and lag in registry data collection means that the most recent studies may not yet be captured. We selected a 2022 cutoff to capture the most recent cost years available in the registry and retained four time periods to balance interpretability and sample size.

Our findings indicate that MFN CEAs apply more stringent value benchmarks than those typically cited in US studies. If these differences reflect tighter budget constraints or higher opportunity costs of health care spending, policymakers should be cautious about adopting pricing policies that would implicitly subject US pharmaceutical spending to benchmarks developed in different institutional and fiscal contexts.

## Conclusion

Although GDP-normalized thresholds have declined overall, they exhibit different patterns in the United States and MFNs. Holding intervention type and disease area constant, US studies continue to cite higher CEA thresholds over time, whereas MFNs have consistently shifted toward lower values. To the extent that lower CEA thresholds in MFNs signal tighter budgetary constraints and other context-specific decision-making, importing these benchmarks into US pricing policy may inadvertently adopt fiscal constraints and valuation norms from fundamentally different health systems.

## Supplementary Material

qxag081_Supplementary_Data

## References

[1] Centers for Medicare & Medicaid Services . CMS announces new drug payment model to strengthen Medicaid and better serve vulnerable Americans. Accessed November 19, 2025. https://www.cms.gov/newsroom/press-releases/cms-announces-new-drug-payment-model-strengthen-medicaid-better-serve-vulnerable-americans

[2] Delivering Most-Favored-Nation Prescription Drug Pricing to American Patients . The White House. Updated May 12, 2025. Accessed October 15, 2025. https://www.whitehouse.gov/presidential-actions/2025/05/delivering-most-favored-nation-prescription-drug-pricing-to-american-patients/

[3] GENEROUS (GENErating cost Reductions fOr U.S. Medicaid) Model | CMS . Accessed November 19, 2025. https://www.cms.gov/priorities/innovation/innovation-models/generous

[4] Philipson TJ, Durie T. The evidence base on the impact of price controls on medical innovation. 2019. Accessed November 18, 2025. https://bfi.uchicago.edu/working-paper/the-evidence-base-on-the-impact-of-price-controls-on-medical-innovation/

[5] Congressional Budget Office. Research and development in the pharmaceutical industry . 2021. https://www.cbo.gov/publication/57025

[6] Long T, Ezell S. The hidden toll of drug price controls: fewer new treatments and higher medical costs for the world. 2023. Accessed November 16, 2025. https://itif.org/publications/2023/07/17/hidden-toll-of-drug-price-controls-fewer-new-treatments-higher-medical-costs-for-world/

[7] Karen Van Nuys P, Darius Lakdawalla P, Dana Goldman P. The elasticity of pharmaceutical innovation: how much does revenue drive new drug development? Published online February 18, 2025. 10.25549/abr5-n176

[8] Kang S, Liu M, Ji Y. Trends in biopharmaceutical clinical trials after medicare drug price negotiation | Health Affairs. Accessed January 11, 2026. https://www.healthaffairs.org/doi/abs/10.1377/hlthaff.2025.00720?journalCode=hlthaff 10.1377/hlthaff.2025.00720 41494116

[9] Zheng H, Patterson JA, Campbell JD. The inflation reduction act and drug development: potential early signals of impact on post-approval clinical trials. Ther Innov Regul Sci. 2025;59(4):781–789. 10.1007/s43441-025-00774-2 40261541 PMC12181196

[10] Neumann PJ, Kim DD. Cost-effectiveness thresholds used by study authors, 1990-2021. JAMA. 2023;329(15):1312–1314. 10.1001/jama.2023.1792 37071104 PMC10114019

[11] Dunn A, Grosse SD, Zuvekas SH. Adjusting health expenditures for inflation: a review of measures for health services research in the United States. Health Serv Res. 2018;53(1):175–196. 10.1111/1475-6773.12612 27873305 PMC5785315

[12] Papanicolas I, Woskie LR, Jha AK. Health care spending in the United States and other high-income countries. JAMA. 2018;319(10):1024–1039. 10.1001/jama.2018.1150 29536101

[13] Lising A, Drummond M, Barry M, Augustovski F. Payers' use of independent reports in decision making—will there be an ICER effect? ISPOR | International Society For Pharmacoeconomics and Outcomes Research. Accessed March 13, 2026. https://www.ispor.org/publications/journals/value-outcomes-spotlight/abstract/march-april-2017/payers-use-of-independent-reports-in-decision-making-will-there-be-an-icer-effect

[14] Dubois RW. "CVS To Restrict Patient Access Using Cost-Effectiveness: Too Much Too Soon" Health Affairs Blog. September 17 2018. 10.1377/forefront.20180913.889578

[15] Kesselheim AS, Avorn J, Sarpatwari A. The high cost of prescription drugs in the United States: origins and prospects for reform. JAMA. 2016;316(8):858–871. 10.1001/jama.2016.11237 27552619

[16] Hunt TL, Luce BR, Page MJ, Pokrzywinski R. Willingness to pay for cancer prevention. PharmacoEconomics. 2009;27(4):299–312. 10.2165/00019053-200927040-00003 19485426

[17] Ben-Aharon O, Iskrov G, Sagy I, Greenberg D. Willingness to pay for cancer prevention, screening, diagnosis, and treatment: a systematic review. Expert Rev Pharmacoecon Outcomes Res. 2023;23(3):281–295. 10.1080/14737167.2023.2167713 36635646

[18] Gopal S, Sharpless NE. Cancer as a global health priority. JAMA. 2021;326(9):809–810. 10.1001/jama.2021.12778 34357387

[19] Claxton K, Martin S, Soares M, et al Methods for the estimation of the NICE cost effectiveness threshold. Health Technol Assess. 2015;19(14):1–504. 10.3310/hta19140 PMC4781395 25692211

[20] Martin S, Lomas J, Claxton K, Longo F. How effective is marginal healthcare expenditure?: new evidence from England for 2003/04 to 2012/13. Appl Health Econ Health Policy. 2021;19:885–903. 10.1007/s40258-021-00663-3 34286470

[21] Martin S, Claxton K, Lomas J, Longo F. How responsive is mortality to locally administered healthcare expenditure? Estimates for England for 2014/15. Appl Health Econ Health Policy. 2022;20(4):557–572. 10.1007/s40258-022-00723-2 35285000

[22] Martin S, Claxton K, Lomas J, Longo F. The impact of different types of NHS expenditure on health: marginal cost per QALY estimates for England for 2016/17. Health Policy. 2023;132:104800. 10.1016/j.healthpol.2023.104800 37004415

[23] Changes to NICE's cost-effectiveness thresholds confirmed . NICE website: The National Institute for Health and Care Excellence. Updated December 1, 2025. Accessed December 18, 2025. https://www.nice.org.uk/news/articles/changes-to-nice-s-cost-effectiveness-thresholds-confirmed

