# Supplementary material for: Cost-effectiveness thresholds used in the United States vs most favored nations
Source: Health Aff Sch. 2026 Apr 3;4(4):qxag081. doi: 10.1093/haschl/qxag081 (PMC13093214; doi:10.1093/haschl/qxag081)
Supplement: qxag081_Supplementary_Data [file qxag081_supplementary_data.zip › Appendix_Thresholds_03232026.docx]

**Appendix**

**Appendix 1. Country-level Analysis**

Table A1-1. Country-level Statistics

| Country | Numbers of Publications | Proportions among the country group | Thresholds Mean (X GDP per capita) |
| --- | --- | --- | --- |
| **Most Favored Nations** | 3388 | 100% | 1.2690 |
| Canada | 698 | 20.6% | 1.5078 |
| Denmark | 93 | 2.75% | 1.1454 |
| France | 192 | 5.67% | 1.6751 |
| Germany | 270 | 7.97% | 1.3629 |
| Italy | 252 | 7.44% | 1.4199 |
| Japan | 193 | 5.70% | 1.358 |
| Switzerland | 84 | 2.48% | 0.9959 |
| United Kingdom | 1606 | 47.4% | 1.1064 |
| **United States** | 3488 | 100% | 2.1032 |

Source: Author’s analysis of published cost-effectiveness analyses (CEAs) from the Tufts Medical Center’s CEA Registry database.

*Note.*

Table A1-1 summarizes the number of cost-per-QALY studies by country. The mean threshold values (excluding missingness) were 1.269X GDP per capita in MFNs and 2.103X GDP per capita in the United States. Across MFNs, the relatively lower mean threshold pattern is consistent across countries.

Table A1-2. Country-specific Logistic Regression of Citing Cost-Effectiveness Threshold above 1X GDP per capita

| Characteristics | Coefficients | SE | Decomposition^(b)^ |
| --- | --- | --- | --- |
| Cancer-related CEAs vs. Non-cancer | 0.8460*** | 0.0892 |  |
| Pharmaceutical Intervention vs. Other | 0.3032*** | 0.0639 |  |
| **Cost Year(Reference: Before 2000)** | | |  |
| 2000 – 2009 | -0.5782* | 0.2288 |  |
| 2010 – 2019 | -1.6234*** | 0.2230 |  |
| Since 2020 | -1.3379*** | 0.2577 |  |
| **Country(Reference: United States)**^(a)^ | | |  |
| Canada | -0.9233*** | 0.1081 | 22.4% |
| Denmark | -1.3530*** | 0.2621 | 4.3% |
| France | -0.5564** | 0.1959 | 3.8% |
| Germany | -0.6126** | 0.1666 | 5.8% |
| Italy | -0.1879 | 0.1692 | 1.6% |
| Japan | -0.1978 | 0.1809 | 1.3% |
| Switzerland | -1.4568*** | 0.2547 | 4.2% |
| United Kingdom | -1.0146*** | 0.0767 | 56.6% |

Source: Author’s analysis of published cost-effectiveness analyses (CEAs) from the Tufts Medical Center’s CEA Registry database.

*Notes.*

1. We report country-level main effects to illustrate cross-country heterogeneity while keeping the table concise.
2. Decomposition: We conducted a weighted decomposition of country-level log-odds coefficients, weighting each country’s effect by its proportion in MFN studies to quantify its contribution to the overall MFN–U.S. difference. The MFN coefficient remained negative and statistically significant in most cases, indicating that the findings are not attributable to any single country.

*: p<0.05; **: p<0.01; ***: p<0.001.

SE = Standard Errors

**Appendix 2. Pharmaceutical-specific statistics**

Table A2. Thresholds Cited in Cost-Effectiveness Analyses with Any Pharmaceutical Interventions

|  | Cost Year  Number of studies (col percentage) | | | | |
| --- | --- | --- | --- | --- | --- |
|  | Before 2000 | 2000 - 2009 | 2010 - 2019 | Since 2020 | All Periods |
| **Most Favored Nations** | 83 | 604 | 904 | 54 | 1645 |
| ≤1X GDP per capita | 6 (7.2) | 120 (19.9) | 353 (39.0) | 25 (46.3) | 504 (30.6) |
| 1-3X GDP per capita | 19 (22.9) | 335 (55.5) | 393 (43.5) | 23 (42.6) | 770 (46.8) |
| >3X GDP per capita | 7 (8.4) | 17 (2.8) | 34 (3.8) | 1 (1.9) | 59 (3.6) |
| Any thresholds | 32 (38.6) | 472 (78.1) | 780 (86.3) | 49 (90.7) | 1333 (81.0) |
| No threshold cited | 51 (61.4) | 132 (21.9) | 124 (13.7) | 5 (9.3) | 312 (19.0) |
| **United States** | 142 | 420 | 799 | 125 | 1486 |
| ≤1X GDP per capita | 4 (2.8) | 47 (11.2) | 165 (20.7) | 11 (8.8) | 227 (15.3) |
| 1-3X GDP per capita | 42 (29.6) | 265 (63.1) | 514 (64.3) | 98 (16.2) | 919 (61.8) |
| >3X GDP per capita | 23 (16.2) | 23 (5.5) | 43 (5.4) | 11(8.8) | 100 (6.7) |
| Any thresholds | 69 (48.6) | 335 (79.8) | 722 (90.4) | 120 (96.0) | 1246 (83.8) |
| No threshold cited | 73 (51.4) | 85 (20.2) | 77 (9.6) | 5(4.0) | 240 (16.2) |

Source: Author’s analysis of published cost-effectiveness analyses (CEAs) from the Tufts Medical Center’s CEA Registry database.

*Note.*

Among CEAs involving pharmaceutical interventions, MFN countries increasingly cite lower thresholds (particularly ≤1X GDP) over time, whereas U.S. studies consistently reference higher thresholds (1–3X GDP), which is consistent with our main analysis across both pharmaceutical and non-pharmaceutical interventions.

**Appendix 3. Inclusion of “No Thresholds Cited”**

We estimated a multinomial logistic regression to examine factors associated with cited cost-effectiveness thresholds in U.S. and MFN-based CEAs, treating “No threshold cited” as a separate category. The dependent variable was categorized as ≤1X GDP per capita, 1-3X GDP per capita, >3X GDP per capita, and no threshold cited. Predictors were the same as in the main analysis, with interaction terms omitted to keep the analysis concise.

Results from this sensitivity analysis are consistent with the main findings.

Table A3. Multinomial Logistic Regression for Cost-Effectiveness Thresholds Cited by the Authors of the US or MFN-based CEAs

| **Predictor** | **Outcome** | **Contrast** | **Estimate** | **SE** | **P** |
| --- | --- | --- | --- | --- | --- |
| Pharmaceutical Intervention | ≤1X GDP | Yes vs. No | -0.05041 | 0.01058 | < 0.001 |
| Pharmaceutical Intervention | 1-3X GDP | Yes vs. No | 0.04299 | 0.01227 | < 0.001 |
| Pharmaceutical Intervention | >3X GDP | Yes vs. No | 0.01328 | 0.00522 | 0.01098 |
| Pharmaceutical Intervention | No threshold | Yes vs. No | -0.00586 | 0.00936 | 0.53091 |
| MFN | ≤1X GDP | Yes vs. No | 0.13249 | 0.01063 | < 0.001 |
| MFN | 1-3X GDP | Yes vs. No | -0.14769 | 0.01229 | < 0.001 |
| MFN | >3X GDP | Yes vs. No | -0.01663 | 0.00517 | 0.0013 |
| MFN | No threshold | Yes vs. No | 0.03184 | 0.00943 | < 0.001 |
| Cancer-related CEAs | ≤1X GDP | Yes vs. No | -0.12261 | 0.01213 | < 0.001 |
| Cancer-related CEAs | 1-3X GDP | Yes vs. No | 0.12501 | 0.01586 | < 0.001 |
| Cancer-related CEAs | >3X GDP | Yes vs. No | 0.02049 | 0.00768 | 0.00762 |
| Cancer-related CEAs | No threshold | Yes vs. No | -0.02289 | 0.01242 | 0.06529 |
| Cost Year | ≤1X GDP | 2000 - 2009 vs. Before 2000 | 0.1068 | 0.01329 | < 0.001 |
| Cost Year | ≤1X GDP | 2010 - 2019 vs. Before 2000 | 0.3007 | 0.01329 | < 0.001 |
| Cost Year | ≤1X GDP | Since 2020 vs. Before 2000 | 0.24408 | 0.02819 | < 0.001 |
| Cost Year | 1-3X GDP | 2000 - 2009 vs. Before 2000 | 0.31634 | 0.02261 | < 0.001 |
| Cost Year | 1-3X GDP | 2010 - 2019 vs. Before 2000 | 0.22613 | 0.02154 | < 0.001 |
| Cost Year | 1-3X GDP | Since 2020 vs. Before 2000 | 0.31608 | 0.03387 | < 0.001 |
| Cost Year | >3X GDP | 2000 - 2009 vs. Before 2000 | -0.0971 | 0.01626 | < 0.001 |
| Cost Year | >3X GDP | 2010 - 2019 vs. Before 2000 | -0.10221 | 0.016 | < 0.001 |
| Cost Year | >3X GDP | Since 2020 vs. Before 2000 | -0.09736 | 0.01863 | < 0.001 |
| Cost Year | No threshold | 2000 - 2009 vs. Before 2000 | -0.32603 | 0.02457 | < 0.001 |
| Cost Year | No threshold | 2010 - 2019 vs. Before 2000 | -0.42462 | 0.0235 | < 0.001 |
| Cost Year | No threshold | Since 2020 vs. Before 2000 | -0.46279 | 0.02764 | < 0.001 |

Source: Author’s analysis of published cost-effectiveness analyses (CEAs) from the Tufts Medical Center’s CEA Registry database.
